# The Role of Human Papilloma Virus (HPV) in Primary Lung Cancer Development: State of the Art and Future Perspectives

**DOI:** 10.3390/life14010110

**Published:** 2024-01-10

**Authors:** Dania Nachira, Maria Teresa Congedo, Ettore D’Argento, Elisa Meacci, Jessica Evangelista, Carolina Sassorossi, Giuseppe Calabrese, Adriana Nocera, Khrystyna Kuzmych, Rosaria Santangelo, Guido Rindi, Stefano Margaritora

**Affiliations:** 1 Department of General Thoracic Surgery, Fondazione Policlinico Universitario “A. Gemelli”, IRCCS, Università Cattolica del Sacro Cuore, 00168 Rome, Italy; mariateresa.congedo@policlinicogemelli.it (M.T.C.); elisa.meacci@policlinicogemelli.it (E.M.); jessica.evangelista@policlinicogemelli.it (J.E.); sassorossi.caro@gmail.com (C.S.); giuseppe93calabrese@virgilio.it (G.C.); adriana.nocera91@gmail.com (A.N.); kkristina.kuz@gmail.com (K.K.); stefano.margaritora@policlinicogemelli.it (S.M.); 2 Medical Oncology, Comprehensive Cancer Center, Fondazione Policlinico Universitario “A. Gemelli”, IRCCS, Università Cattolica del Sacro Cuore, 00168 Rome, Italy; ettore.dargento@policlinicogemelli.it; 3 Institute of Microbiology, Fondazione Policlinico Universitario “A. Gemelli”, IRCCS, Università Cattolica del Sacro Cuore, 00168 Rome, Italy; rosaria.santangelo@policlinicogemelli.it; 4 Institute of Pathology, Fondazione Policlinico Universitario “A. Gemelli”, IRCCS, Università Cattolica del Sacro Cuore, 00168 Rome, Italy; guido.rindi@policlinicogemelli.it

**Keywords:** human papilloma virus (HPV), primary lung cancer, HPV-related cancers, cervical cancer, oropharyngeal cancer, immunotherapy

## Abstract

Non-small cell lung cancer (NSCLC) is the leading cause of cancer-related mortality worldwide. Notably, the incidence of lung cancer among never-smokers, predominantly women, has been rising in recent years. Among the various implicated risk factors, human papilloma virus (HPV) may play a role in the development of NSCLC in a certain subset of patients. The prevalence of high-risk HPV-DNA within human neoplastic lung cells varies across the world; however, the carcinogenetic role of HPV in NSCLC has not been completely understood. Bloodstream could be one of the routes of transmission from infected sites to the lungs, along with oral (through unprotected oral sex) and airborne transmission. Previous studies reported an elevated risk of NSCLC in patients with prior HPV-related tumors, such as cervical, laryngeal, or oropharyngeal cancer, with better prognosis for HPV-positive lung cancers compared to negative forms. On the other hand, 16% of NSCLC patients present circulating HPV-DNA in peripheral blood along with miRNAs expression. Typically, these patients have a poorly differentiated NSCLC, often diagnosed at an advanced stage. However, HPV-positive lung cancers seem to have a better response to target therapies (EGFR) and immune checkpoint inhibitors and show an increased sensitivity to platinum-based treatments. This review summarizes the current evidence regarding the role of HPV in NSCLC development, especially among patients with a history of HPV-related cancers. It also examines the diagnostic and prognostic significance of HPV, investigating new future perspectives to enhance cancer screening, diagnostic protocols, and the development of more targeted therapies tailored to specific cohorts of NSCLC patients with confirmed HPV infection.

## 1. Introduction

Lung cancer is the second most diagnosed cancer in the world [1] and the leading cause of cancer-related mortality (18% of all cancer-related deaths). Recently, the incidence of lung cancer among never-smokers, predominantly women, has been rising, resulting in 17,000–26,000 deaths solely in the United States [2].

Therefore, a better understanding of the mechanisms behind carcinogenesis in non-small cell lung cancer (NSCLC) and its prevention is fundamental. While tobacco remains the primary etiological factor, other risk factors include occupational exposure to carcinogens, genetic mutations, air pollution, and viral infections. Among viruses, high-risk human papilloma viruses (HPV) 16 and 18 show a major ability for tissue adherence, persistent infection, and the integration of their genic fragments into the host genome. 

Syrjänenin [3], in 1979, first suggested the potential involvement of HPV in NSCLC development. Subsequently, several retrospective studies have reported the detection of HPV in lung cancer cells, with an incidence varying around the world, with the highest recorded in Asia (40.3%) [4,5,6].

HPV-DNA has been identified not only in human neoplastic lung cells [4,5,6] but also in serum, plasma, and peripheral blood mononuclear cells [6]. The bloodstream itself could be one of the ways of transmission from infected organs to the lungs, together with oral (via unprotected oral sex) and airborne routes [6].

Previous studies reported an increased risk of NSCLC in patients with prior HPV-related tumors, such as vulvar, cervical, laryngeal, or oropharyngeal cancers [7,8,9].

Consequently, HPV might be an etiological factor for NSCLC development, particularly among patients with a history of HPV-related cancers. Notably, it may work synergistically with tobacco smoke in the carcinogenetic process. Smoke facilitates the transition towards the squamous metaplasia of pulmonary epithelium, where the presence of squamocolumnar junctions aids HPV entry [5], as in the cervical cancer process. 

Squamous cell carcinoma (44% of NSCLC cases in men and 25% in women) [1,2] stands as the lung cancer histology with the highest prevalence of HPV infection (up to 48.1%) [5,6,10,11]. This prevalence is often associated with smoking habits [5]. In contrast, adenocarcinoma (28% of NSCLC cases in men and 42% in women) [1,2] demonstrates a reported HPV prevalence of 44.4% [5,6,10,11]. Adenocarcinoma is the main histotype found in non-smokers, displaying totally different molecular characteristics and expressing the highest number of driver mutations within the lung cancer landscape. 

Despite the fact that, in recent years, enough data were collected regarding the prevalence of HPV infection among NSCLC patients [6], the carcinogenetic role of HPV infection in lung cancer is still unclear and debated. Moreover, its diagnostic, prognostic, and therapeutic value in HPV-positive NSCLC remains unexplored.

In this scenario, this review aims to summarize the latest evidence regarding the role of HPV in NSCLC development, particularly among patients with a history of HPV-related cancers. It also examines the diagnostic and prognostic significance of HPV, investigating new future perspectives in order to improve cancer screening and diagnostic protocols and to develop more effective and tailored NSCLC therapies, in specific cohorts of patients.

## 2. The Role of HPV in Lung Cancer Development

### 2.1. HPV Genotypes, Integration Sites in Host Genome, and Oncogenetic Activity

HPV is a small (52–55 nm), non-enveloped, icosahedral, double-stranded DNA virus (about 8000 base pairs), containing three main regions: early (E genes), late (L genes), and long control region (LCR) [12]. There exist over 170 described HPV types [13], and among them, “high-risk” HPVs (16, 18, 31, 33, 39, 45, 51, 52, and 58) are responsible for persistent infections that significantly increase the risk of cancer compared to “low-risk” HPV variants (6, 11, 42, 43, and 44). Cervical cancer is associated with HPV16 and 18 infections in 70% of cases [14], while head and neck cancer mainly with HPV16 infection [15] (25% of cases due to sexually transmitted HPV). 

In more than 80% of cervical cancers, HPV is often found integrated into the host DNA, a process intricately linked to HPV-induced oncogenesis [16]. In fact, HPV16-positive cancers show virus integration in 76% of cases, whereas the integration is consistently evident in HPV18-positive forms [16].

Two integration schemes are described: the first type involves a single copy of HPV-DNA integration into cellular DNA, while the second type entails multiple tandem head-to-tail repeats of virus genome integrated in a single host genic locus [17].

**Figure 1:**
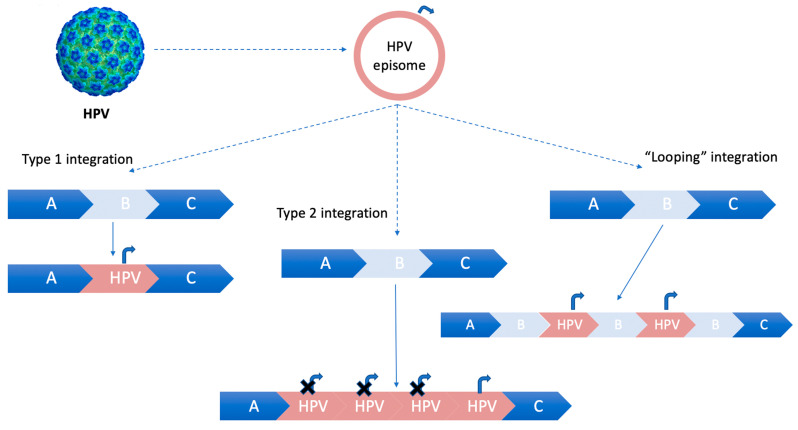
HPV integration models for integrating into the host genome. Curved arrow (Pe, early promoter). Type 1 integration: a single copy of HPV episomal DNA is integrated into the host genome. Type 2 integration: multiple tandem head-to-tail repeats of HPV-DNA are integrated into a single host DNA locus. Type 3 integration: “looping” integration of HPV-DNA, without losing flanking host sequences. The concatamers of host and virus genomes are amplified in tandem in several copies and then reinserted into the host genome.

In case the integration of HPV-DNA into host chromosomes does not lead to a loss of the flanking host sequences, a “looping” integration model occurs. This model involves the amplification of concatamers of host and virus genomes in tandem in several copies, which are subsequently reinserted into the host genome [18], as illustrated in Figure 1.

The “looping” model of HPV integration is a widely accepted mechanism [18].

Integration in human genome often happens within some specific target sequences, called “hotspots”, found in regions characterized by fragile sites and active transcription.

**Figure 2:**
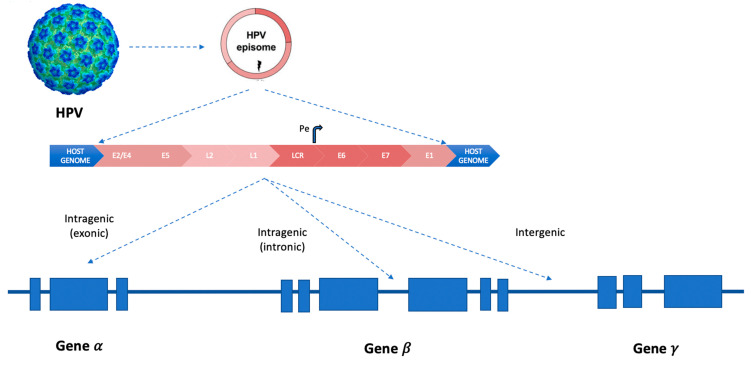
HPV integration patterns in human genes inside chromosomes. Curved arrow (Pe, early promoter). LCR (long control region). The three different types of integration models of HPV-DNA in “hotspots” sites of the human genome: intra-genic (inside exons or introns) and inter-genic.

In squamous cell carcinomas, more than 300 HPV integration types or signatures were described (inter- or intra-genic, Figure 2), varying according to the involved HPV genotype [19]. 

The MACROD2, MIPOL1/TTC6, and TP63 are the most common integration sites, as well as MYC, TMEM49, FANCC and RAD51B genes28–30, POU5F1B, FHIT, KLF12, KLF5, HMGA2, LRP1B, LEPREL1, DLG2, and SEMA3D (less common integration sites were reported in the following genes: AGTR2, DMD, CDH7, DCC, HS3ST4, CPNE8, C9orf85, MSX2, and CADM2.9) [20,21,22]. 

In the early stages of the HPV life cycle, after integrating its genome into the host genome, it overexpresses two primary oncoproteins called E6 and E7. These proteins inactivate two tumor suppression proteins: E6 disrupts p53, while E7 affects the retinoblastoma protein. By modifying the host cellular cycle, these actions favor viral genome amplification and transcription processes [23]. Other target proteins and genes regulated by E6/E7 are as follows: HIF-1α, VEGF, IL-6, IL-10, Mcl-1, Bcl-2, cIAP-2, EGFR, FHIT, hTERT, HER- 2, ROS1, and AhR. The collective impact of these alterations generally enhances lung cell proliferation, angiogenesis, and cell immortalization [6,11,24]. In particular, hypoxia-inducible factor-1α (HIF-1α) protein accumulation and vascular endothelial growth factor (VEGF) and interleukin-8 (IL-8) expression promotes angiogenesis [24]. Interleukin-10 (IL-10), secreted by macrophages, interferes with host immune response, establishing a persistent infection and starting tumorigenesis [2]. Additionally, Bcl-2 and cIAP-2 prevent apoptosis, contributing to tumor development [24]. E6 oncoprotein causes oxidative stress within cells (through increased ROS levels), leading to DNA damage and an enhanced formation of 8-OH-dG (8-hydroxy-2′deoxyguanosine), which may trigger epidermal growth factor (EGFR) mutation [24]. Oncoproteins also upregulate interleukin-6 (IL-6) that promotes Mcl-1, through phosphatidylinositol-3-OH kinase (P13K), thereby increasing tumor malignancy [24]. E7 can interfere with aryl hydrocarbon pathway (AhR), a ligand-activated transcription factor, resulting in cell cycle blockade [24]. However, its precise mechanism has not been deciphered in detail yet.

Furthermore, the overexpression of E6/E7 oncogene can be triggered by viral genome hypermethylation (through hypermethylated CpG in E2 binding sites) [25]. Moreover, E6/E7 themselves can stimulate DNA methyltransferase activity, thereby increasing the methylation levels within infected cells, a process that contributes to their transformation towards a neoplastic state [25]. 

### 2.2. HPV Infection of Lung Cells: Pathogenesis, Prevalence, and Carcinogenesis

Particularly for lung cancer, nine high-risk HPV genotypes were recently detected in patients with NSCLC, i.e., 16, 18, 31, 35, 45, 51, 52, 56, and 59 [26], and often multiple HPV types were concurrently observed in lung tissues [26]. The most frequently detected HPV genotypes were 16, 18, and 56 [27,28,29,30]. 

HPV-DNA was discovered not only in neoplastic lung cells [4,5,6,11,27] but also in human blood [6,11,27] and peripheral mononuclear cells [6]. The integrated form of HPV-DNA was identified in tissue samples of patients affected by NSCLC, while the episomal and mixed forms were identified in blood samples [26]. In 2023, Sun et al. used the Mendelian randomization to provide extensive analysis on the causal effect of HPV in cancer development [31]. Mendelian randomization is an epidemiological method to analyze the casual relationship between HPV exposure and outcomes by using single nucleotide polymorphisms [31]. Using this method on a large HPV16/17 protein exposure dataset, the authors demonstrated how HPV18 E7 exposure posed to be a risk factor for NSCLC development [26]. In a very recent and valid meta-analysis, Drokow et al. [11] showed that patients infected with HPV type 16 were at a higher risk for NSCLC development compared to those infected with type 18 (OR = 1.95, 95% CI: 1.00–3.79). They also concluded that the risk of NSCLC post-HPV infection was higher in squamous cell carcinoma patients (OR = 1.62, 95% CI: 0.62–4.29), smokers (OR = 1.09, 95% CI: 0.74–1.61), and patients over 55 years of age (OR = 1.09, 95% CI: 0.74–1.61).

**Figure 3:**
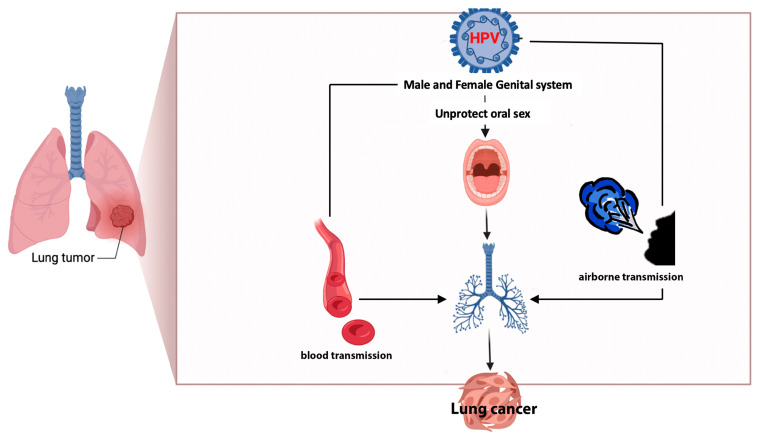
Possible ways of the transmission of HPV to lung parenchyma.

The high concordance among studies regarding the presence of HPV types (16/18) in lung cells, peripheral blood, and cervical cancer [32] implicated the potential transfer of the same virus from one site to another. This observation suggests the possibility of bloodstream transmission, which could serve as a route of dissemination [8,33] alongside oral (through unprotected oral sex) and airborne transmission [6,32] (through exhaled air), Figure 3.

**Figure 4:**
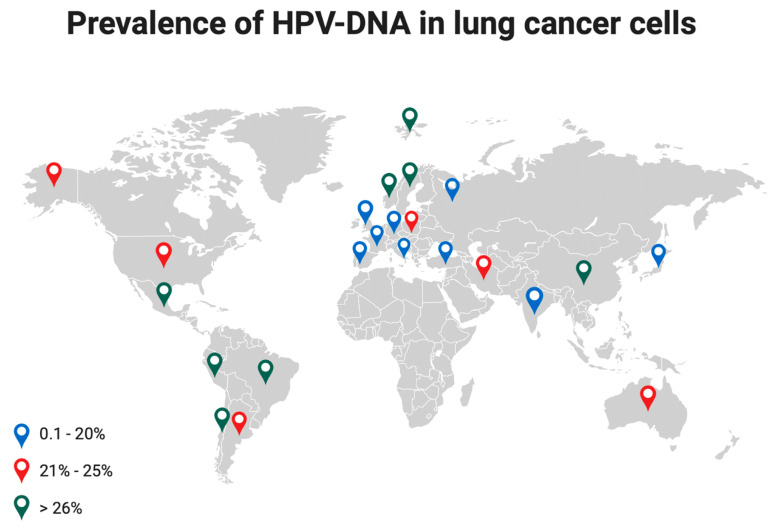
Prevalence of HPV-DNA in lung cancer cells at different latitudes. Colored dots indicate a different range of HPV prevalence.

The detection of high-risk HPV16 and 18 in lung cancer cells varies based on geographic areas [6] with the highest incidence observed in Asia (40.3%), followed by Latin America (33.6%), Europe (25.6%), North America (15.4%), Japan (16.9%) [34], and Russia (12.7%) [26]. In Italy, HPV prevalence in NSCLC ranges between 0 and 21.1% [6], Figure 4.

The HPV infection rates vary not only across continents but also among the different histological types [11], with a higher presence of HPV-DNA in NSCLC than in normal lung tissues (OR (95% CI) = 5.38 (3.21–9.00), *p* < 0.0001) [6,35,36,37].

Indeed, for squamous cell carcinoma, the reported infection rates were 46.3%, 21.3%, and 32.2% in Asia, Europe, and America, respectively [11]. In contrast, for adenocarcinoma, the infection rates were 21.2%, 9.5%, and 10.5% in Asia, Europe, and America, respectively [11]. According to some authors, the variations in HPV prevalence among studies might be due to the use of different detection methods [11]. And, about that, the PCR techniques on lung tissues should be considered the preferred method for the detection of HPV proteins due to their higher sensitivity, while serological methods should be viewed as alternative approaches [11].

Usually, the most common diagnosed histological type in non-smoker patients affected by NSCLC is adenocarcinoma. It is characterized by a higher prevalence of targetable driver mutations, such as epidermal growth factor receptor mutations (EGFRm) and human epidermal growth factor receptor-2 mutations (HER2m), as well as anaplastic lymphoma kinase (ALK) and ROS proto-oncogene 1 (ROS1) translocations [36].

However, squamous cell carcinoma seems to have the highest correlation (25.8%) with HPV infection (OR = 9.78, 95% CI = 6.28–15.22, *p* < 0.001) [33] compared to adenocarcinoma (21.1%) [5,6]. The authors of [5] have noted a higher, though statistically not significant, association between smokers or former smokers and the prevalence of HPV in their lung cancer cells. This is likely related to the role of tobacco smoking in the histological transition of pulmonary epithelium to squamous metaplastic cells, facilitated by the presence of squamocolumnar junctions (SCJ). These SCJs serve as the preferential entry pathway for HPV, as in cervical cancer [5]. Consequently, smoke and HPV infection, driven by the expression of viral oncoproteins, may have a synergetic effect on lung cancer development [8,11]. Indeed, smoking can decrease the number of Langhans cells (antigen presenting cells of the epithelial tissue), creating a form of immune deficiency [11]. Furthermore, smoking reduces the immune response by inducing the release of IL-4, prompting the release of T-helper (Th2) cells. This increase in Th2 presence favors the microbial colonization of the lung [38].

Additionally, Peña et al. [39] demonstrated that tobacco smoke can activate HPV16 early promoter (p97) in the lung epithelium, consequently leading to the overexpression of E6/E7 oncoproteins through the PI3K, Akt, mTOR pathway. This correlation between HPV and tobacco smoke exacerbates DNA damage of lung epithelial cells [39].

HPV can also function as an independent carcinogenetic factor in non-smoker patients [8]. Specifically, E6 suppresses the interaction between p53 and DDX3, resulting in the inactivation of p21. Consequently, the cyclin A/CDK2 complex phosphorylates pRb, enhancing cell proliferation, releasing E2F transcription factor, triggering G1/S transition in cell cycle, and inhibiting apoptosis [40]. The HDAC/pRb/E2F complex releases HDAC, after the interaction with pRb, that causes the hypermethylation of p16INK4 and tumor progression. VEGF, IL-8, and HIF-1alpha promote angiogenesis. Then, E7- and E6-inactivated p53 induce antiapoptosis through the upregulation of Mcl-1 by the PI3K/akT-(IL-6)-(IL-17) pathway [11,26]. The same mechanism could explain the higher incidence of HPV16 and HPV18 infection in lung adenocarcinoma with EGFR mutations, as shown by Harabajsa et al. [30] in their series and meta-analysis. In fact, E6 antiapoptosis functioning through the PI3K/akT/EGFR pathway may explain the NSCLC tumorigenesis in patients with EGFR gene mutation and HPV infection [30].

Furthermore, E6 increases Bcl-2 levels and cisplatin resistance [41] and promotes the expression of PDL-1 through ERK-C/EBPβ-TLR4-NF-κB, fostering lung cancer invasiveness [42]. 

Additionally, previous studies have shown that HPV can also act through a “hit and run” mechanism, being involved in the initial phases of carcinogenesis before being cleared by the cells when no longer required [43,44].

Moreover, HPV amplifies the expression and activity of proangiogenic MMP-2 and MMP-9, by inducing IL-8 [36]. It hyperactivates the signal transducers and activators of the transcription 3 (STAT3) signaling pathway [36]. 

STAT3 enhances tumorigenesis by regulating various target genes and epithelial-mesenchymal transition (EMT; the process where epithelial cells lose their cell polarity and cell–cell adhesion, becoming mesenchymal stem cells, with migratory and invasive properties) in lung cancer cells [36,45]. In particular, HPV16 E6/E7 oncoproteins can upregulate the phosphorylation levels of STAT3, activating STAT3 signaling and inducing EMT in NSCLC [36,45]. The Pi3K/AkT/HIF-1α signaling pathway might also contribute to the progression of HPV-associated NSCLC by mediating the hypoxemia-induced EMT and EMT-related transcription factors [38].

In 2023, Nie et al. [28] also demonstrated how the interaction between long noncoding RNA SNHG1 (whose expression is increased in NSCLC cells by HPV16 E6) and EGFR can activate a downstream pathway that promotes tumor angiogenesis and VEGF (vascular endothelial growth factor) expression.

Furthermore, the decreased expression of LKB1 mRNA, coupled with the overexpression of E6 and E7 oncoproteins [36], and the increased levels of HIF1 and VEGF gene expression [36] (that regulate inflammation and antitumoral immune response) promote cell proliferation. 

In 2021, Hussen et al. [45] demonstrated, for the first time, how HPV infection and the interplay between gene products (E6 and E7) and cellular microRNAs (miRNAs; small non-coding RNAs of approximately 22 nucleotides) are involved in EMT, as a basis of cancer development. The HPV-DNA was found in 51.4% of NSCLC cells (28.6% were integrated form, 5.3% episomal form, and 66.1% mixed form), predominantly HPV type 16 was found in 41.1% of cases. The highest levels of viral oncoproteins E6 and E7 corresponded with the advanced tumor stage [45], associated with particular miRNAs that interact with EMT-related genes. Recently, the authors of [26] also inferred the role of HPV infection in lung cancer development through inflammation and EMT. Indeed, they observed a significantly increased level of inflammatory cytokines in HPV-positive lung cancers compared to negative forms [26].

Moreover, in a recent and very interesting study, the researchers from the Crick Institute found EGFR and KRAS driver mutations in 15% and 53% of 247 normal lung tissue samples, respectively. These driver mutations in EGFR and KRAS genes commonly occur with aging and seem to promote carcinogenesis in lung cells, presenting these mutations when exposed to air pollution. Specifically, air pollution might enhance macrophage response, elevating the inflammatory mediator interleukin-1β, thereby promoting carcinogenesis in mutated EGFR bronchial epithelium [46]. 

**Figure 5:**
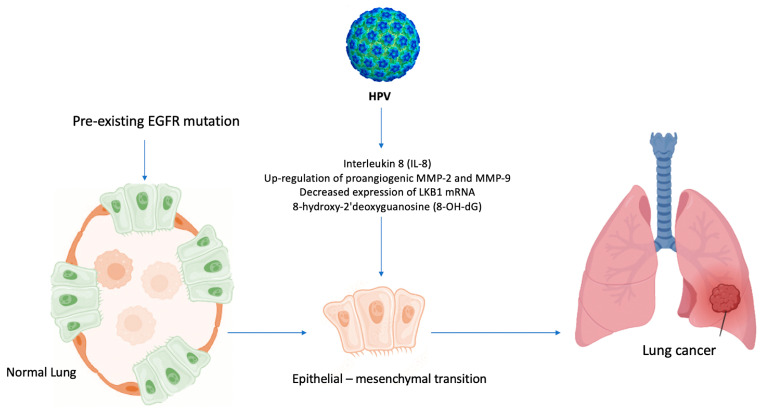
Hypothesis of HPV-induced carcinogenesis in never-smokers. HPV enhances the formation of 8-OH-dG (8-hydroxy-2′deoxyguanosine) and the levels of IL-8, upregulates MMP-2 and MMP-9, and decreases the expression of LKB1 mRNA, leading to EMT and activating epidermal growth factor (EGFR) mutations in never-smoker lung cells.

In light of the above-mentioned evidence [3,27,45], a similar mechanism, based on inflammation induced by air pollution [46], could explain the role of HPV in lung carcinogenesis among never-smoker patients, Figure 5.

A recent study revealed that 16% of NSCLC patients presented circulating HPV-DNA in peripheral blood [47] and abnormally expressed miRNAs in HPV-associated tumors [47]. The patients with circulating HPV-DNA positively presented poorly differentiated NSCLC (mainly adenocarcinoma) and were diagnosed in an advanced stage compared to those without circulating HPV-DNA [47]. However, the carcinogenetic role of circulating HPV-DNA in NSCLC has not been demonstrated yet. In particular, the combined detection of miR-210 and miR-144 in plasma had the highest predictive value to detect a positive circulating HPV-DNA patient (AUC 0.938, 95% CI: 0.871–0.976, *p* < 0.001), with a sensitivity of 93.75% and a specificity of 94.05%. The overexpression of miR-182 and miR-183 (upregulated in HPV-related cancers) seemed to be associated with poor prognosis and metastatic diseases in NSCLC [47]. MiR-210, known as “micromanager of the hypoxic pathway”, has carcinogenic effects, and it was upregulated in NSCLC patients, while miR-144, with a tumor-suppressor function, was usually downregulated [47].

### 2.3. Second Primary Lung Cancer in Previous HPV-Related Tumors

In this context, the occurrence of second primary lung cancer following prior HPV-related tumors deserves a special discussion.

Indeed, cancer survivors appeared to have a 14% higher risk of developing a second primary tumor compared to the general population [9]. Eight percent of survivors in the United States developed a second primary malignancy (SPLC), which in 25% of cases was a SPLC (the most recorded second primary cancer) [9]. 

The analysis of Surveillance, Epidemiology and End Results (SEER) Program data, from 1992 to 2008, revealed that head and neck cancer survivors had a higher risk of developing a SPLC. In particular, 10% of patients with a previous laryngeal cancer developed a SPLC at their 10-year follow-up [9]. A prior study on SEER data from 1973 to 1992 recorded a 5% incidence of SPLC in patients who had survived head and neck cancers [47]. This was considered a “field effect” of carcinogenesis in tobacco smokers. The median time for the onset of SPLC after a previous cancer was 42 months in this subset of patients, with an overall poor survival rate (median survival: 8 months) [48]. 

Conversely, it has been reported that patients with a previous HPV-related oropharyngeal cancer had a lower risk of developing a tobacco-related second primary neoplasia (5.9%) than patients with HPV-negative oropharyngeal cancers (26.9%) [49] that had a 10.4-fold higher risk (CI 95%: 2.4–45.3, *p*: 0.002).

Furthermore, a higher 5-year survival rate was reported among patients with a tobacco-related second primary neoplasm following prior HPV-positive oropharyngeal cancers (97.6%) compared to previous HPV-negative cancers (62.0%), *p* < 0.001 [49]. 

To the best of our knowledge, no data about the prevalence of HPV-positive SPLC in previous positive oropharyngeal cancers are available in the literature.

On the other hand, in women with a previous HPV-related urogenital cancer, a higher incidence of SPLC was recorded [8]. Henning et al. [8] showed that 49% of SPLC cases were HPV positive within a cohort of 75 patients previously affected by SPLC following HPV-positive cervical intraepithelial neoplasia (CIN III). In particular, the histology of HPV-positive SPLC was as follows: 35% adenocarcinoma, 30% squamous cell carcinoma, 22% oat cell carcinoma, 5% large cell carcinoma, 3% anaplastic carcinoma, 3% low-differentiated carcinoma, and 3% malignant cylindroma. And the main HPV types involved in carcinogenesis were as follows: HPV16 (48%), HPV6 (24%), HPV16/6 (24%), and HPV18 (4%).

### 2.4. Diagnostic Tools to Discriminate Lung Metastases from SPLCs

Therefore, in patients with a prior history of head/neck or cervical carcinoma, it is critical to distinguish between lung metastasis and an SPLC in case of development of a lung tumor.

However, while it is easy to discern if the histology of a second primary malignancy is different from a primary HPV-related cancer, sometimes, when the histology is the same, it may be challenging to distinguish a second primary malignancy from a metastatic cancer [48]. 

In such cases, the decision is often based on clinical experience, radiological evaluation (the shape of the lesion), and the oncological history of the patient (the stage of primary cancer and time intercourse between the first and the second cancer).

Research on the expression of viral onco-protein RNAs (E6 and E7) through sensitive RNA scope technology (RNA in situ hybridization), along with p16 (an inhibitor of cyclin-dependent kinases, that slows down the progression of cell cycle from G1 phase to S phase) may help in the diagnostic process.

In fact, it was already demonstrated that the overexpression of P16 is strongly related to HPV infection [50], being used as an immunohistochemical marker for cervical [51] and oropharyngeal HPV-related carcinoma [52]. 

A useful tool to differentiate primary from metastatic lung cancer can be HPV typing, a method used routinely for cervical cancer, as proposed by Weichert et al. [53].

In their study, the authors of [53] tested 26 patients with head and neck or cervical cancers, along with concurrent or subsequent lung tumor, to determine their HPV status. They used polymerase chain reaction (PCR), following DNA extraction from tissues (using a modified protocol of the QIAamp DNA mini Kit procedure, QIAGEN, Hilden, Germany), and array analysis (the HPV type was identified with LCD- Array HPV Type 3.5C, Chipron, GmbH, berlin, Germany). In those patients, where an identical status was found between primary HPV-related cancer and concomitant or subsequent lung cancer, a pulmonary metastatic disease was suggested. Conversely, a discordant HPV status leaned towards an independent SPLC diagnosis. Interestingly, with this method, the authors of [53] found 50% (5 out of 10) of lung lesions, initially classified as metastases from neck and head tumors, that were identified as SPLC due to the presence of discordant HPV status.

Furthermore, the HPV integration site/signature within the hot genome, together with integration model [19,54], particularly in cancer cells, may help in better differentiating primary lung cancer from metastatic lesion in patients with previous HPV-related tumors. For instance, a distinct HPV integration site in lung cancer cells compared to that in previous HPV-related cancer may suggest an SPLC diagnosis, indicating a new carcinogenic process of the same HPV genotype in pulmonary cells. Conversely, the presence of the same HPV type with matching signature in the host genome would suggest the metastatic origin of the lung tumor.

The recent advancement of next-generation sequencing (NGS) for both RNA and DNA has allowed the precise determination of virus integration loci within the host genome and genomic rearrangements in those sites, improving the understanding of viral integration process and carcinogenesis [16].

Also, Campbell et al. [55] demonstrated how by using new tools for integrating omics data with molecular taxonomy, it is possible to sub-classify squamous cell carcinoma from different sites into different molecular subtypes by analyzing recurrent alterations in chromosomes, DNA methylation, miRNAs, and mutations.

Notably, the most frequent HPV integration sites often coincide with concomitant methylated CpG sites. Therefore, the detection of a specific combination of methylated CpG sites (nt5606, nt5609, nt5615, nt5378, etc.) can be used as potential diagnosis biomarkers for squamous cell carcinomas.

### 2.5. Prognostic Aspects of HPV Infection in NSCLC

As for other HPV-related cancers [56], HPV infection seems to be associated with better clinical outcomes in NSCLC, in terms of better response to therapies and overall survival [33]. Specifically, patients affected by an HPV16/18-positive lung cancer have significantly higher survival rates compared to those of HPV16/18-negative lung cancer patients [33].

In 2022, Rojas et al. [36] evaluated for the first time, in a retrospective study on 133 Latin American IV-stage NSCLC patients (26% HPV+), the correlation between clinical outcomes and HPV infection, which was detected not only by HPV-DNA positiveness but also by the determination of viral activity through the expression of E6/mRNA oncoproteins. They found significant improvements in response rate (82.4% compared to 47.1% in negative ones) and overall survival (2-year overall survival was more than 25% higher in HPV-positive forms compared to negative ones, *p*: 0.008) for immune checkpoint inhibitors in HPV-positive cases.

The underlying reasons behind the favorable prognosis associated with HPV infection in NSCLC remain unknown. A possible explanation could be derived from a recent study (October 2022), where Wang et al. [57] showed how HPV immortalizes cancer cells not only by inhibiting tumor suppressors (Rb and p53) but also by activating the EGFR pathway. According to their results, HPV16E6/18E6 and EGFR expression in NSCLC correlates with a greater survival rate in older lung adenocarcinoma patients without brain metastasis, with a smoking history, and with wild-type EGFR status. Additionally, this group of patients demonstrated an increased sensitivity to cisplatin-based chemotherapy due to the action of HPV16E5/16E6/16E7 oncoproteins on EGFR nuclear trafficking [57]. Other data available in the literature further support these observations, showing better prognosis, higher survival rates [58], and longer metastatic-free survival [26] in HPV18- and 19-positive NSCLCs compared to negative forms.

### 2.6. New Frontiers in NSCLC Treatments

Prior to the first report by Wang et al. [57] in 2022, the role of HPV lung infection in the aggressiveness of lung cancer was completely unexplored. However, the growing evidence supporting a better prognosis for HPV-positive NSCLC has pushed to consider HPV status as an important biomarker in the therapeutic pathway of these patients. Due to a lack of evidence until recent relevant studies were published, testing for HPV status of lung cancer cells was only performed in research settings and was not a part of routine clinical practice [32,56,57].

The increased sensitivity to platinum-based therapies [57] and radiotherapy [56], enhanced response to target therapy (EGFR) [57], and improved outcomes with immune checkpoint inhibitors [6] in HPV-positive NSCLC represent significant advancements. These findings underscore the potential of HPV infection detection as a prognostic and predictive factor, especially in patients treated with the latest therapeutic approaches.

Thus, “personalized treatment protocols” could be considered for lung cancer patients and should be extended beyond common genetic mutations, cancer profiling, and immunophenotype to HPV oncoprotein status [57].

## 3. Future Perspective

In the near future, new promising developments will be possible in the landscape of lung cancer. 

First of all, the integration of omics information with molecular data, such as chromosome alterations, DNA methylation, and mutations, is expected to lead to a refined molecular subclassification of NSCLC that will improve targeted therapies.

Secondly, new tools are poised to improve the diagnosis and the treatment of specific patient cohorts, especially those with HPV-positive NSCLC. 

As viral load of HPV is a useful predictor for cervical cancer development [59,60,61]. The viral load in lung cells could be a prognostic marker [33] for NSCLC development in the future. But no data have been published on the specific topic yet. However, several other biomarkers have been already evaluated and look promising for improving NSCLC screening programs.

For instance, as reported above, circulating HPV-DNA-positive patients have a higher possibility to present with an advanced poorly differentiated lung adenocarcinoma at the time of diagnosis compared to circulating HPV-DNA-negative patients [47]. These patients express specific cellular miRNAs, such as miR-210, miR-182, miR-183, and miR-144, which could serve as novel biomarkers for identifying circulating HPV-DNA-positive NSCLC patients [47]. Moreover, miR-182 and miR-183 could be considered biomarkers for poor prognosis in NSCLC patients [47].

HPV infection significantly increases DNA damage and mutations as inflammation-induced oxidative stress supports lung adenocarcinoma development. Indeed, the levels of the oxidative stress biomarker 8-OH-dG are closely associated with EGFR mutation in lung cancer [62] and with the grade of dysplasia in HPV-associated cervical carcinogenesis [63], Figure 5. Thus, these findings may be the basis for developing new immunological and molecular preventive approaches, particularly for non-smokers at risk of lung cancer, including patients harboring EGFR mutations and who are HPV positive.

In the next years, NGS is poised to offer new insights into virus integration processes and carcinogenesis, potentially unveiling novel targets for silencing transcriptionally active HPV domains. This may represent the first and fundamental step towards developing new targeted therapies against HPV-related cancers. 

Another important aspect to be evaluated in the near future would be that of HPV vaccination.

Vaccination has shown to prevent over 90% of HPV infections, which in turn reduces the risk of HPV-related cancers development; thus, it may also be useful for reducing the risk of NSCLC according to some authors [11,32].

However, it is known that HPV prophylactic vaccination does not provide effective protection to patients already infected with the virus [64]. Once the virus integrates into the host genome, many early (E1, E2, E3, and E4) and late genes (L1 and L2) are lost, making preventive vaccination ineffective against HPV-related cancer such as NSCLC [64]. Moreover, considering that vaccines against HPV are available since 2006 (Gardasil^®^4, a quadrivalent vaccine available in 2006, Cervarix™, a bivalent vaccine, in 2007, and Gardasil^®^9, a nonavalent vaccine, in 2014 [65]), this may explain why a certain age-group of patients, already infected by high-risk HPV and at risk to develop HPV-related NSCLC, may not benefit from the positive effects of the vaccination campaign, in our opinion. Furthermore, the vaccination adhesion rate is not the same around the world due to the lack of government founding or political support. All these factors might be the basis of the apparent discrepancy between the declining trend of certain high-risk HPV genotypes reported after national widespread vaccination [66] and the increasing prevalence of NSCLC, particularly among young patients [67].

Further research is essential to understand the potential impact of HPV vaccination in reducing the incidence of lung cancer.

In the future, another weapon against HPV-related cancers could be therapeutic vaccination.

Available therapeutic vaccines against HPV are designed to target E6/7 oncoproteins and aim to boost cellular immunity and improve body’s response to cancer treatments [65]. 

Briefly, these therapeutic vaccines primarily work by stimulating adaptive T cell immunity, in particular naïve T cells to produce cytotoxic T lymphocytes (CTL) against HPV infected cells, and by inducing CD4+T cells to produce cytokines and antigen-presenting cells (APC). The main therapeutic vaccines are based on the following: live vectors (bacterial and viral), peptides, proteins, liposomes, nuclei acids (DNA and mRNA), and whole cells (dendritic cells or tumor cells). At present, though not yet available for clinical use, these vaccines are being rigorously tested in clinical trials for the treatment of cervical cancer, demonstrating safety and good tolerance levels [65]. 

## 4. Conclusions

High-risk HPV infection, in particular, HPV16 and 18, appear to increase the risk of NSCLC development. However, HPV-positive lung cancers seem to have a better prognosis compared to negative forms and an improved response to target therapies (EGFR), immune checkpoint inhibitors, and platinum-based chemotherapy. 

Further studies are necessary to clarify the pathogenesis, the oncogenesis, and the prognostic role of HPV infection in lung cancer. Indeed, a deeper understanding of HPV’s potential role within the complex carcinogenetic mechanisms of NSCLC could improve the prevention and the better comprehension of the most diffused cancer in the world in specific patient cohorts. 

Moreover, in the near future, a deeper comprehension of HPV carcinogenic role in NSCLC development could be relevant for adopting efficient HPV-targeted prevention strategies. This could lead to advancements in NSCLC screening, the development of cost-effective diagnostic protocols (through research on peripheral blood HPV-DNA and miRNAs), and the tailored implementation of immune-based and target therapies for subsets of NSCLC patients with HPV infection (such as those with a history of HPV-related cervix or oropharynx cancers).

## References

[1] Sung H., Ferlay J., Siegel R.L., Laversanne M., Soerjomataram I., Jemal A., Bray F. (2021). Global Cancer Statistics 2020: Globocan Estimates of Incidence and Mortality Worldwide for 36 Cancers in 185 Countries. CA Cancer J. Clin..

[2] Corrales L., Rosell R., Cardona A.F., Martín C., Zatarain-Barrón Z.L., Arrieta O. (2020). Lung cancer in never smokers: The role of different risk factors other than tobacco smoking. Crit. Rev. Oncol. Hematol..

[3] Syrjänen K.J. (1979). Condylomatous changes in neoplastic bronchial epithelium. Rep. A Case Respir..

[4] He F., Xiong W., Yu F., Xiao R., Ye H., Li W., Liu Z., Hu Z., Cai L. (2020). Human papillomavirus infection maybe not associated with primary lung cancer in the Fujian population of China. Thorac. Cancer.

[5] de Oliveira T.H.A., do Amaral C.M., de França São Marcos B., Nascimento K.C.G., de Miranda Rios A.C., Quixabeira D.C.A., Muniz M.T.C., Silva Neto J.D.C., de Freitas A.C. (2018). Presence and activity of HPV in primary lung cancer. J. Cancer Res. Clin. Oncol..

[6] Tsyganov M.M., Pevzner A.M., Ibragimova M.K., Deryusheva I.V., Litviakov N.V. (2019). Human papillomavirus and lung cancer: An overview and a meta-analysis. J. Cancer Res. Clin. Oncol..

[7] Bjørge T., Hennig E.M., Skare G.B., Søreide O., Thoresen S. (1995). Second primary cancers in patients with carcinoma in situ of the uterine cervix. The Norwegian experience 1970–1992. Int. J. Cancer.

[8] Hennig E.M., Suo Z., Karlsen F., Holm R., Thoresen S., Nesland J.M. (1999). HPV positive bronchopulmonary carcinomas in women with previous high-grade cervical intraepithelial neoplasia (CIN III). Acta Oncol..

[9] Donin N.M., Kwan L., Lenis A.T., Drakaki A., Chamie K. (2019). Second primary lung cancer in United States Cancer Survivors, 1992–2008. Cancer Causes Control.

[10] Srinivasan M., Taioli E., Ragin C.C. (2009). Human papillomavirus type 16 and 18 in primary lung cancers—A meta-analysis. Carcinogenesis.

[11] Drokow E.K., Effah C.Y., Agboyibor C., Budu J.T., Arboh F., Kyei-Baffour P.A., Xiao Y., Zhang F., Wu I.X. (2023). Microbial infections as potential risk factors for lung cancer: Investigating the role of human papillomavirus and chlamydia pneumoniae. AIMS Public Health.

[12] Working Group on the Evaluation of Carcinogenic Risks to Humans (2007). Human Papillomaviruses. Lyon (FR): International Agency for Research on Cancer. (IARC Monographs on the Evaluation of Carcinogenic Risks to Humans, No. 90.) 1, Human Papillomavirus (HPV) Infection. https://www.ncbi.nlm.nih.gov/books/NBK321770/.

[13] Bzhalava D., Guan P., Franceschi S., Dillner J., Clifford G. (2013). A systematic review of the prevalence of mucosal and cutaneous human papillomavirus types. Virology.

[14] Ault K.A. (2006). Epidemiology and natural history of human papillomavirus infections in the female genital tract. Infect. Dis. Obstet. Gynecol..

[15] Parkin D.M. (2006). The global health burden of infection-associated cancers in the year 2002. Int. J. Cancer.

[16] McBride A.A., Warburton A. (2017). The role of integration in oncogenic progression of HPV–Associated cancers. PLoS Pathog..

[17] Jeon S., Allen-Hoffmann B.L., Lambert P.F. (1995). Integration of human papillomavirus type 16 into the human genome correlates with a selective growth advantage of cells. J. Virol..

[18] Groves I.J., Coleman N. (2018). Human. papillomavirus genome integration in squamous carcinogenesis: What have next-generation sequencing studies taught us?. J. Pathol..

[19] Kamal M., Lameiras S., Deloger M., Morel A., Vacher S., Lecerf C., Dupain C., Jeannot E., Girard E., Baulande S. (2021). Human papilloma virus (HPV) integration signature in Cervical Cancer: Identification of MACROD2 gene as HPV hot spot integration site. Br. J. Cancer.

[20] Zhang R., Shen C., Zhao L., Wang J., McCrae M., Chen X., Lu F. (2016). Dysregulation of host cellular genes targeted by human papillomavirus (HPV) integration contributes to HPV-related cervical carcinogenesis. Int. J. Cancer.

[21] Zhang Y., Koneva L.A., Virani S., Arthur A.E., Virani A., Hall P.B., Warden C.D., Carey T.E., Chepeha D.B., Prince M.E. (2016). Subtypes of HPV-positive head and neck cancers are associated with HPV characteristics, copy number alterations, PIK3CA mutation, and pathway signatures. Clin. Cancer Res..

[22] Koneva L.A., Zhang Y., Virani S., Hall P.B., McHugh J.B., Chepeha D.B., Wolf G.T., Carey T.E., Rozek L.S., Sartor M.A. (2018). HPV integration in HNSCC correlates with survival outcomes, immune response signatures, and candidate drivers. Mol. Cancer Res..

[23] Münger K., Howley P.M. (2002). Human papillomavirus immortalization and transformation functions. Virus Res..

[24] de Freitas A.C., Gurgel A.P., de Lima E.G., de França São Marcos B., do Amaral C.M. (2016). Human papillomavirus and lung cancinogenesis: An overview. J. Cancer Res. Clin. Oncol..

[25] von Knebel Doeberitz M., Prigge E.S. (2019). Role of DNA methylation in HPV associated lesions. Papillomavirus Res..

[26] Syganov M.M., Ibragimova M.K., Rodionov E.O., Cheremisina O.V., Miller S.V., Tuzikov S.A., Litvyakov N.V. (2023). Human Papillomavirus in Non-Small Cell Lung Carcinoma: Assessing Virus Presence in Tumor and Normal Tissues and Its Clinical Relevance. Microorganisms.

[27] Osorio J.C., Candia-Escobar F., Corvalán A.H., Calaf G.M., Aguayo F. (2022). High-Risk Human Papillomavirus Infection in Lung Cancer: Mechanisms and Perspectives. Biology.

[28] Nie Z., Zhang K., Li Z., Bing X., Jin S., Li M. (2023). Human papillomavirus 16 E6 promotes angiogenesis of lung cancer via SNHG1. Cell Biochem. Biophys..

[29] Liu J., Huang B., Xiu Z., Zhou Z., Liu J., Li X., Tang X. (2018). PI3K/Akt/HIF-1α signaling pathway mediates HPV-16 oncoprotein-induced expression of EMT-related transcription factors in non-small cell lung cancer cells. J. Cancer.

[30] Harabajsa S., Šefčić H., Klasić M., Milavić M., Židovec Lepej S., Grgić I., Zajc Petranović M., Jakopović M., Smojver-Ježek S., Korać P. (2023). Infection with human cytomegalovirus, Epstein-Barr virus, and high-risk types 16 and 18 of human papillomavirus in EGFR-mutated lung adenocarcinoma. Croat. Med. J..

[31] Sun J., Xiang J., An Y., Xu J., Xiong Y., Wang S., Xia Q. (2023). Unveiling the Association between HPV and Pan-Cancers: A Bidirectional Two-Sample Mendelian Randomization Study. Cancers.

[32] Li Y.J., Tsai Y.C., Chen Y.C., Christiani D.C. (2009). Human papilloma virus and female lung adenocarcinoma. Semin. Oncol..

[33] Zhai K., Ding J., Shi H.Z. (2015). HPV and lung cancer risk: A meta-analysis. J. Clin. Virol..

[34] Hasegawa Y., Ando M., Kubo A., Isa S., Yamamoto S., Tsujino K., Kurata T., Ou S.H., Takada M., Kawaguchi T. (2014). Human papilloma virus in non-small cell lung cancer in never smokers: A systematic review of the literature. Lung Cancer.

[35] Ragin C., Obikoya-Malomo M., Kim S., Chen Z., Flores-Obando R., Gibbs D., Koriyama C., Aguayo F., Koshiol J., Caporaso N.E. (2014). HPV-associated lung cancers: An international pooled analysis. Carcinogenesis.

[36] Rojas L., Mayorga D., Ruiz-Patiño A., Rodríguez J., Cardona A., Archila P., Avila J., Bravo M., Ricaurte L., Sotelo C. (2022). Human papillomavirus infection and lung adenocarcinoma: Special benefit is observed in patients treated with immune checkpoint inhibitors. ESMO Open.

[37] Karnosky J., Dietmaier W., Knuettel H., Freigang V., Koch M., Koll F., Zeman F., Schulz C. (2021). HPV and lung cancer: A systematic review and meta-analysis. Cancer Rep..

[38] Burger M.P., Hollema H., Gouw A.S., Pieters W.J., Quint W.G. (1993). Cigarette smoking and human papillomavirus in patients with reported cervical cytological abnormality. BMJ.

[39] Peña N., Carrillo D., Muñoz J.P., Chnaiderman J., Urzúa U., León O., Tornesello M.L., Corvalán A.H., Soto-Rifo R., Aguayo F. (2015). Tobacco smoke activates human papillomavirus 16 p97 promoter and cooperates with high-risk E6/E7 for oxidative DNA damage in lung cells. PLoS ONE.

[40] Szymonowicz K.A., Chen J. (2020). Biological and clinical aspects of HPV-related cancers. Cancer Biol. Med..

[41] Tung M.C., Lin P.L., Cheng Y.W., Wu D.W., Yeh S.D., Chen C.Y., Lee H. (2016). Reduction of microRNA-184 by E6 oncoprotein confers cisplatin resistance in lung cancer via increasing Bcl-2. Oncotarget.

[42] Chen M.J., Wang Y.C., Wang L., Shen C.J., Chen C.Y., Lee H. (2022). PD-L1 expressed from tumor cells promotes tumor growth and invasion in lung cancer via modulating TGF-β1/SMAD4 expression. Thorac. Cancer.

[43] Viarisio D., Müller-Decker K., Accardi R., Robitaille A., Dürst M., Beer K., Jansen L., Flechtenmacher C., Bozza M., Harbottle R. (2018). Beta HPV38 oncoproteins act with a hit-and-run mechanism in ultraviolet radiation-induced skin carcinogenesis in mice. PLoS Pathog..

[44] Kostov S., Dzhenkov D., Metodiev D., Kornovski Y., Slavchev S., Ivanova Y., Yordanov A. (2021). A case of human papillomavirus infection and vulvar cancer in a young patient—“hit and run” theory. Gynecol. Oncol. Rep..

[45] Hussen B.M., Ahmadi G., Marzban H., Fard Azar M.E., Sorayyayi S., Karampour R., Nahand J.S., Hidayat H.J., Moghoofei M. (2021). The role of HPV gene expression and selected cellular MiRNAs in lung cancer development. Microb. Pathog..

[46] Swanton C., Hill W., Lim E., Lee C., Weeden C., Augustine M., Chen K., Kuan F.-C., Marongiu F., Rodrigues F. (2022). Mechanism of Action and an Actionable Inflammatory Axis for Air Pollution Induced Non-Small Cell Lung Cancer: Towards Molecular Cancer Prevention. ESMO Congress 2022, LBA 1. Ann. Oncol..

[47] Wu Y., Yin Q., Zhou Y.L., He L., Zou Z.Q., Dai X.Y., Xia W. (2021). Evaluation of microRNAs as potential biomarkers in circulating HPV-DNA-positive non-small cell lung cancer patients. Cancer Biol. Ther..

[48] Jayaprakash V., Cheng C., Reid M., Dexter E.U., Nwogu C.E., Hicks W., Sullivan M., Demmy T.L., Yendamuri S. (2011). Previous head and neck cancers portend poor prognoses in lung cancer patients. Ann. Thorac. Surg..

[49] Martel M., Alemany L., Taberna M., Mena M., Tous S., Bagué S., Castellsagué X., Quer M., León X. (2017). The role of HPV on the risk of second primary neoplasia in patients with oropharyngeal carcinoma. Oral. Oncol..

[50] Chang S.Y., Keeney M., Law M., Donovan J., Aubry M.C., Garcia J. (2015). Detection of human papillomavirus in non-small cell carcinoma of the lung. Hum. Pathol..

[51] Cioffi-Lavina M., Chapman-Fredricks J., Gomez-Fernandez C., Ganjei-Azar P., Manoharan M., Jorda M. (2010). P16 expression in squamous cell carcinomas of cervix and bladder. Appl. Immunohistochem. Mol. Morphol..

[52] Oguejiofor K.K., Hall J.S., Mani N., Douglas C., Slevin N.J., Homer J., Hall G., West C.M. (2013). The prognostic significance of the biomarker p16 in oropharyngeal squamous cell carcinoma. Clin. Oncol..

[53] Weichert W., Schewe C., Denkert C., Morawietz L., Dietel M., Petersen I. (2009). Molecular HPV typing as a diagnostic tool to discriminate primary from metastatic squamous cell carcinoma of the lung. Am. J. Surg. Pathol..

[54] Liu L., Ying C., Zhao Z., Sui L., Zhang X., Qian C., Wang Q., Chen L., Guo Q., Wu J. (2018). Identification of reliable biomarkers of human papillomavirus 16 methylation in cervical lesions based on integration status using high-resolution melting analysis. Clin. Epigenetics..

[55] Campbell J.D., Yau C., Bowlby R., Liu Y., Brennan K., Fan H., Taylor A.M., Wang C., Walter V., Akbani R. (2018). Genomic, Pathway Network, and Immunologic Features Distinguishing Squamous Carcinomas. Cell Rep..

[56] Hussain S.S., Lundine D., Leeman J.E., Higginson D.S. (2021). Genomic Signatures in HPV-Associated Tumors. Viruses.

[57] Wang J.L., Lee W.J., Fang C.L., Hsu H.L., Chen B.J., Liu H.E. (2022). Human Papillomavirus Oncoproteins Confer Sensitivity to Cisplatin by Interfering with Epidermal Growth Factor Receptor Nuclear Trafficking Related to More Favorable Clinical Survival Outcomes in Non-Small Cell Lung Cancer. Cancers.

[58] Wang J.L., Fang C.L., Wang M., Yu M.C., Bai K.J., Lu P.C., Liu H.E. (2014). Human papillomavirus infections as a marker to predict overall survival in lung adenocarcinoma. Int. J. Cancer..

[59] Wu Y., Chen Y., Li L., Yu G., Zhang Y., He Y. (2006). Associations of high-risk HPV types and viral load with cervical cancer in China. J. Clin. Virol..

[60] Gravitt P.E., Kovacic M.B., Herrero R., Schiffman M., Bratti C., Hildesheim A., Morales J., Alfaro M., Sherman M.E., Wacholder S. (2007). High load for most high risk human papillomavirus genotypes is associated with prevalent cervical cancer precursors but only HPV16 load predicts the development of incident disease. Int. J. Cancer.

[61] Huang Y., Huang M.N., Li N., Li X.G., Wu L.Y. (2008). Association between human papillomavirus DNA load and development of cervical intraepithelial neoplasia and cervical cancer. Int. J. Gynecol. Cancer.

[62] Kawahara A., Azuma K., Hattori S., Nakashima K., Basaki Y., Akiba J., Takamori S., Aizawa H., Yanagawa T., Izumi H. (2010). The close correlation between 8-hydroxy-2′-deoxyguanosine and epidermal growth factor receptor activating mutation in non-small cell lung cancer. Hum. Pathol..

[63] Romano G., Sgambato A., Mancini R., Capelli G., Giovagnoli M.R., Flamini G., Boninsegna A., Vecchione A., Cittadini A. (2000). 8-hydroxy-2′-deoxyguanosine in cervical cells: Correlation with grade of dysplasia and human papillomavirus infection. Carcinogenesis.

[64] zur Hausen H. (2002). Papillomaviruses and cancer: From basic studies to clinical application. Nat. Rev. Cancer..

[65] Mo Y., Ma J., Zhang H., Shen J., Chen J., Hong J., Xu Y., Qian C. (2022). Prophylactic and Therapeutic HPV Vaccines: Current Scenario and Perspectives. Front. Cell Infect. Microbiol..

[66] Lin Y., Lin W.Y., Lin T.W., Tseng Y.J., Wang Y.C., Yu J.R., Chung C.R., Wang H.Y. (2023). Trend of HPV Molecular Epidemiology in the Post-Vaccine Era: A 10-Year Study. Viruses.

[67] Ganti A.K., Klein A.B., Cotarla I., Seal B., Chou E. (2021). Update of Incidence, Prevalence, Survival, and Initial Treatment in Patients With Non-Small Cell Lung Cancer in the US. JAMA Oncol..

